# The Aetiology of Delay to Commencement of Adjuvant Chemotherapy following Colorectal Resection

**DOI:** 10.1155/2014/670212

**Published:** 2014-03-17

**Authors:** G. S. Simpson, R. Smith, P. Sutton, A. Shekouh, C. McFaul, M. Johnson, D. Vimalachandran

**Affiliations:** ^1^Countess of Chester Hospital NHS Foundation Trust, Countess of Chester Health Park, Liverpool Road, Chester CH2 1UL, UK; ^2^University of Liverpool, Liverpool, Merseyside L69 3BX, UK

## Abstract

*Purpose*. Timely administration of adjuvant chemotherapy following colorectal resection is associated with improved outcome. We aim to assess the factors which are associated with delay to adjuvant chemotherapy in patients who underwent colorectal resection as part of an enhanced recovery protocol. *Method*. A univariate and multivariate analysis of patient data collected as part of a prospectively maintained database of colorectal cancer patients between 2007 and 2012. *Results*. 166 patients underwent colorectal resection followed by adjuvant chemotherapy. Median postoperative hospital stay was 6 days, and time to commencement of adjuvant chemotherapy was 50 days. Longer inpatient stay correlated with increased time to adjuvant chemotherapy (*P* = 0.05). Factors found to be independently associated with duration of hospital stay and time to commencement of adjuvant chemotherapy included stoma formation (*P* = 0.032), anastaomotic leak (*P* = 0.027), and preoperative albumin (*P* = 0.027). The use of laparoscopic surgery was associated with shorter time to adjuvant chemotherapy but did not reach significance (*P* = 0.143). *Conclusion*. A number of independent variables associated with delay to adjuvant therapy previously not described have been identified. Further work may be required to elucidate the effect that these variables have on long-term outcome.

## 1. Introduction

Colon and rectal cancer is a common malignancy worldwide, having the third highest incidence of all cancers with around 1 million diagnoses worldwide each year [1]. Multimodality treatment strategies are employed in the management of colorectal malignancy; with neoadjuvant and adjuvant treatments complimenting the mainstay of treatment-surgical resection.

The use of adjuvant chemotherapy (AC) following surgical resection of colorectal cancer has been shown to improve outcome [2–5]. Adjuvant chemotherapy has been advocated in patients with stage II disease associated with adverse disease features including T4 disease, perforation or obstruction [6], and in all patients with stage III disease [7].

The timing of administration of adjuvant chemotherapy following surgical resection has been proposed as a factor that potentially affects overall outcome, although this has not been proven conclusively. Some studies have demonstrated that initiation of chemotherapy occurring more promptly following surgical resection is being associated with improved outcome [8–10]. A meta-analysis found poorer outcomes if chemotherapy is administered 8 weeks or more after surgery [11], whilst another meta-analysis has reported a decrease in overall survival of 14% for each 4-week delay in administration of adjuvant chemotherapy [12].

Multiple factors dictating postoperative course and outcome in colorectal cancer have been identified including markers of the extent of systemic inflammatory response such as the Glasgow Prognostic Score (GPS), C-reactive protein, and albumin [13–15]. In addition, physiological parameters [16], patient comorbidity [17], and operative strategy [18, 19] have been shown to influence postoperative course and outcome. In contrast, limited information regarding the factors associated with increased delay to commencement of adjuvant therapy is available; however, age and race have been linked to delay in administration of adjuvant chemotherapy [20], whilst the occurrence of surgical complications has been associated with complete omission of adjuvant chemotherapy rather than delay of commencement [21].

Our aim is to identify factors which are associated with increased delay in administration of adjuvant chemotherapy in a cohort of patients undergoing curative resection for colorectal cancer.

## 2. Patients and Methods

An analysis of a prospectively maintained database containing details of all patients undergoing colorectal cancer resections from 2007 to 2012 was performed. All those with stages II-III colorectal cancer who received adjuvant chemotherapy following surgical resection were identified and included in the study. Relevant data pertaining to patient characteristics, operative strategy, complications, histology, biochemical parameters, and adjuvant therapy were extracted and analysed.

### 2.1. Outcome Measures

The time period (days) between surgical resection and commencement of adjuvant chemotherapy was calculated. Pre- and postoperative variables, histology, and biochemical parameters were analysed as to know their influence on the time to administration of adjuvant chemotherapy.

### 2.2. Statistical Analysis

All continuous data were analysed with median, interquartile range, and 95% confidence intervals. Nonparametric tests were employed for comparative purposes (Mann-Whitney *U* test). The interval between surgery and commencement of adjuvant chemotherapy was analysed as time to event data using Cox regression to analyse continuous and categorical variables for univariate and multivariate analysis. Software used included StatView V5 (SAS Institute, Cary, NC).

## 3. Results

166 patients who underwent intended curative resection for colorectal adenocarcinoma followed by adjuvant chemotherapy were identified.  Table 1 outlines the patient demographics and operative details for this patient cohort.  Table 2 outlines the histological characteristics of the resected cancers. Preoperative blood was typically recorded within 24 hours of surgery—median time interval = 1 day (IQR = 1 to 6).

### 3.1. Postoperative Data

The median duration of hospital stay was 6 (IQR = 5 to 8) days. Five patients (3%) had a postoperative anastomotic leak; four of whom required further surgery. Two patients (1%) had significant postoperative bleeding; one of whom required reoperation and one required readmission. The median time interval from hospital discharge to commencing chemotherapy was 50 (IQR = 41 to 58) days. Patients with a longer postoperative inpatient stay exhibited a significant trend towards having a longer time interval from discharge to chemotherapy (linear regression; *t* = 1.94; *P* = 0.050)

### 3.2. Interval from Operation to Chemotherapy

**Figure 1:**
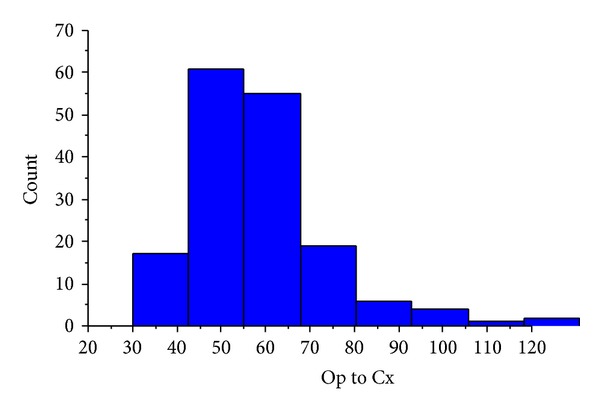
Distribution of time intervals from operation to commencement of chemotherapy (for inclusion in online publication only).

Overall, the median time interval from the date of surgery to date of commencing adjuvant chemotherapy was 58 days (IQR = 39 to 77).  Figure 1 illustrates this distribution. No patients received chemotherapy within 30 days of surgery. 107 (64%) patients received chemotherapy between 30 and 60 days of surgery and 59 (36%) patients received chemotherapy after 60 days.

**Figure 2:**
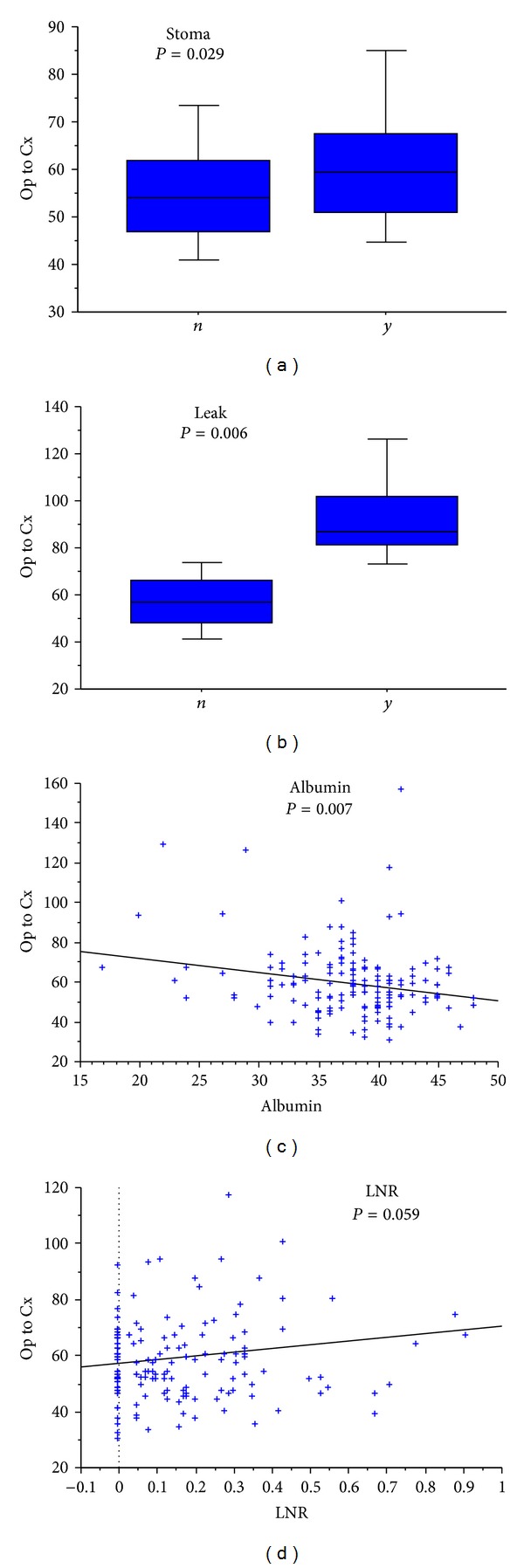
Relationship between requirement of stoma (a), anastomotic leak (b), preoperative serum albumin (c), and lymph node ratio (d) with time to adjuvant chemotherapy (categorical variables = Mann-Whitney; continuous variable = linear regression).

Table 3 demonstrates the relationship between the clinicopathological factors investigated and time from surgery to commencement of adjuvant chemotherapy (Cox regression). From this analysis preoperative hypoalbuminaemia, anastomotic leak, requirement for stoma, and increasing lymph node ratio were all identified as having a potential association with a longer wait to commencement of adjuvant chemotherapy (*P* < 0.100). Patients undergoing laparoscopic surgery exhibited a trend towards shorter time intervals to starting adjuvant chemotherapy but this failed to reach significance (*P* = 0.143). On multivariate Cox regression, all four factors were independently significant.  Figure 2 illustrates the associations between these four variables and time to chemotherapy. Due to incomplete preoperative biochemical data in 7 cases, the final multivariate analysis included 159 patients.

### 3.3. Duration of Hospital Admission

**Figure 3:**
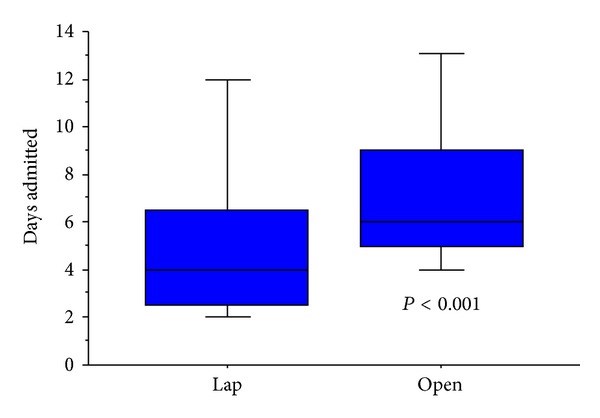
Association between laparoscopic surgery and shorter postoperative stay.

**Figure 4:**
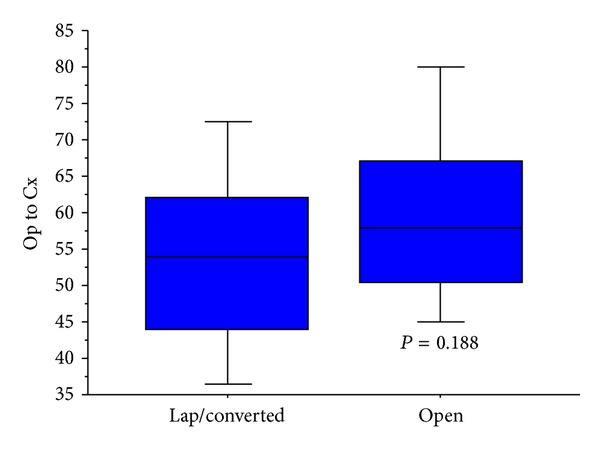
Association between approach to resection and time to adjuvant chemotherapy, laparoscopic/converted versus open (for inclusion in online publication only).

All four variables identified from the above analysis were also found to demonstrate a significant association with increased duration of postoperative stay (Table 4). Alongside this, patients undergoing laparoscopic resections were found to have a shorter postoperative hospital stay than those undergoing open surgery (Mann-Whitney; *P* < 0.001)—Figures 3 and 4.

## 4. Discussion and Conclusion

Adjuvant chemotherapy is a key component in the treatment of colorectal cancer and is shown to improve survival [3–5]. Data assessing the effect of timing of adjuvant chemotherapy have shown an increased mortality in patients where administration of chemotherapy has been delayed beyond 60 days [10, 22]. Only a small number of reports have demonstrated little effect of the timing of adjuvant chemotherapy following colorectal cancer resection on outcome [23, 24]. Recent meta-analyses have shown the benefit of early administration of chemotherapy, demonstrating a decrease in survival of 14% with every 4-week increase in delay to chemotherapy following resection [11, 12]. The finding of improved outcome with timely administration of adjuvant chemotherapy has also been documented in patients with cancer at other sites, most notably the breast [25–27] and pancreas [28].

Our data has identified multiple independently significant factors which are associated with increased delay to provision of adjuvant chemotherapy. Preoperative serum albumin has been shown to be inversely correlated with delay to commencement of adjuvant therapy. Similarly, our data has demonstrated that low preoperative serum albumin is associated with increased postoperative hospital stay. Albumin has previously been identified as a valuable preoperative marker linked to outcome following colorectal resection; however, no accounts are available in the literature showing it to be linked to timely receipt of adjuvant chemotherapy [13–15]. Preoperative albumin represents a potential marker of disease severity which could represent the degree of disease progression relating to operative difficulty and extension of recovery time, detrimentally affecting timely administration of chemotherapy. Additionally, albumin acts as an indicator of poor preoperative nutritional repleteness and overall systemic upset, factors which will dictate postoperative recovery and readiness for adjuvant chemotherapy.

Anastomotic leak following colorectal resection has a profound impact upon postoperative course and has a known detrimental effect on recurrence and overall survival [29, 30], in addition to being associated with significant morbidity and often permanent stoma formation [31]. From our data, it can be seen that this impacts the duration of inpatient stay and its effect extends to timely administration of chemotherapy, with those patients experiencing an anastomotic leak further jeopardized by a delay in the commencement of their systemic therapy.

The use of a defunctioning stoma following colorectal resection has been associated with extended inpatient hospital stay and delay to chemotherapy in this patient population. Available literature shows the formation of a defunctioning stoma to carry morbidity in the early postoperative period [32, 33] and to extend postoperative hospital stay [34], mirroring our findings; however, the presence of a defunctioning stoma being associated with delay to adjuvant therapy has not been previously documented. The use of a defunctioning stoma is commonly associated with patients who have undergone preoperative chemoradiotherapy, rectal resections, and major or difficult resections, factors which may represent the cause of delay as opposed to the presence of a stoma.

Lymph node ratio defined as the number of involved lymph nodes divided by the total nodal yield has been proposed as a valuable prognostic indicator in colorectal cancer with studies showing that poorer long-term outcome is associated with an increasing lymph node ratio [35, 36] potentially as a result of more aggressive tumour biology in those tumours with higher lymph node ratios. Lymph node ratio in this study may represent those patients who have required a more extensive surgical dissection or are more systemically unwell as a consequence of their more aggressive malignancy and thus are more likely to experience a greater time to commencement of adjuvant chemotherapy.

Whilst the effect of delayed chemotherapy has been investigated, research into the aetiology of such a delay has been minimal. In the available evidence, factors which have previously been cited as having an association with increased delay to commencement of chemotherapy include advanced age, patient comorbidity, tumour grade, marital status, postoperative stay, and race [20, 22]. Our findings are not consistent with these previously documented associations. Administration of neoadjuvant therapies, tumour characteristics, patient comorbidity, age, and sex do not have a significant effect on the timely provision of adjuvant therapy in our data.

This study has identified a number of independently significant variables which are associated with delay to administration of adjuvant chemotherapy. The variables identified have not previously been described in the literature. Interestingly, the use of a laparoscopic approach to colorectal resection has been seen to yield a shorter wait to commencement of chemotherapy, although this did not achieve significance. This represents a potential further advantage of laparoscopic surgery in addition to shorter inpatient stay, postoperative pain, cost-effectiveness, and recovery time previously described [37–39]. This study is potentially limited by the retrospective nature of the data analysis.

The importance of timely administration of adjuvant chemotherapy following surgical resection has been identified as of importance in a number of specialties and its benefit has been made evident in colorectal cancer. Vigilance regarding prompt administration of adjuvant chemotherapy to colorectal cancer patients following surgical resection should be promoted, with colorectal teams providing this aspect of treatment as promptly as possible following surgical resection. Our study demonstrates a number of factors associated with delay in receiving adjuvant chemotherapy and may be used to identify patients who are at risk of delayed adjuvant chemotherapy so that this may be addressed in preoperative and intraoperative treatment decisions.

## Figures and Tables

**Table 1:** Patient demographics and operative details.

Number of patients identified	**166**
Gender: men	112 (67%)
Age: median (IQR)	66 (61 to 73) years
Comorbidity	91 (55%)
BMI: median (IQR)	27.3 (24.2 to 30.3)
Neoadjuvant chemoradiotherapy	
None	124 (75%)
Long course	36 (22%)
Short course	6 (3%)
Operation details	
Anterior resection	63 (38%)
Right hemicolectomy	50 (30%)
Left/sigmoid colectomy	30 (18%)
Abdominoperineal resection	13 (8%)
Hartmann's procedure	6 (4%)
Subtotal colectomy	3 (2%)
Panproctocolectomy	1 (<1%)
Mode of surgery	
Open	124 (75%)
Laparoscopic	42 (19%)
Converted	10 (6%)
Elective : emergency	153 (92%) : 13 (8%)
Stoma required	72 (43%)
Preoperative bloods: median (IQR)	
Haemoglobin (g/dL)	13.0 (11.9 to 14.3)
Platelets (×10^6^/mL)	249 (218 to 333)
Neutrophils (×10^6^/mL)	4.6 (3.6 to 5.7)
Lymphocytes (×10^6^/mL)	1.5 (1.0 to 2.0)
Albumin (mg/L)	38 (35 to 41)
C-reactive protein (mg/L)	3 (2 to 11)

IQR: interquartile range.

**Table 2:** Histological tumour characteristics.

*Histology *	
Tumour size: median (IQR)	35 (27–50) mm
Differentiation	
Well/moderate	146 (88%)
Poor	13 (8%)
Complete response	7 (4%)
Node status	
N0	57 (34%)
N1	72 (43%)
N2	37 (22%)
Median nodal yield	14 (10 to 20)
Median number of involved nodes	3 (1 to 5)
Median lymph node ratio	0.18 (0.10 to 0.33)
T stage	
T0/T1	12 (7%)
T2	14 (8%)
T3	92 (55%)
T4	48 (29%)
Resection margin status	
R0	160 (94.4%)
R1	6 (3.6%)
Vascular invasion	
Positive	46 (28%)
Negative	120 (72%)

**Table 3:** Cox regression analysis of factors associated with time from surgery to commencement of adjuvant chemotherapy.

	Univariate analysis			Multivariate analysis (*n* = 159)		
	Hazard ratio (95% CI)	*χ* ^2^	*P*	Hazard ratio (95% CI)	*χ* ^2^	*P*
Age	0.993 (0.978 to 1.007)	0.973	0.324			
Gender (F)	1.310 (0.937 to 1.832)	2.500	0.114			
BMI	0.991 (0.961 to 1.023)	0.292	0.589			
Comorbidity (Y)	1.083 (0.789 to 1.488)	0.247	0.619			
Neoadjuvant therapy (Y)	0.889 (0.625 to 1.265)	0.425	0.514			
Laparoscopic procedure (Y)	1.337 (0.906 to 1.973)	2.141	0.143			
Stoma (Y)	0.757 (0.555 to 1.031)	3.115	**0.078**	0.704 (0.512 to 0.970)	4.613	**0.032**
Tumour size	1.002 (0.994 to 1.010)	0.180	0.672			
Differentiation (poor versus well/moderate)	1.201 (0.679 to 2.122)	0.396	0.529			
T stage	1.009 (0.863 to 1.179)	0.013	0.911			
N stage	0.940 (0.680 to 1.299)	0.141	0.707			
Lymph node ratio	0.494 (0.215 to 1.131)	2.785	0.095	0.408 (0.168 to 0.992)	3.915	**0.048**
Resection margin status (+)	0.917 (0.546 to 1.541)	0.107	0.744			
Vascular invasion (+)	1.151 (0.816 to 1.623)	0.645	0.422			
Anastomotic leak (Y)	0.304 (0.123 to 0.749)	6.701	**0.010**	0.352 (0.140 to 0.887)	4.907	**0.027**
Preoperative haemoglobin	1.006 (0.915 to 1.105)	0.013	0.908			
Preoperative platelets	1.000 (0.999 to 1.002)	0.613	0.434			
Preoperative neutrophils	0.977 (0.915 to 1.042)	0.518	0.472			
Preoperative lymphocytes	0.966 (0.824 to 1.131)	0.187	0.666			
Preoperative C-reactive protein	0.998 (0.994 to 1.001)	1.751	0.186			
Preoperative albumin	1.036 (1.007 to 1.067)	5.933	**0.015**	1.034 (1.004 to 1.065)	4.916	**0.027**
Tumour site (colon versus rectum)	1.092 (0.802 to 1.487)	0.311	0.577			
Emergency (Y)	0.755 (0.426 to 1.339)	0.925	0.336			

**Table 4:** Multivariate Cox regression analysis of hypoalbuminaemia, anastomotic leak, requirement for stoma, lymph node ratio, and association with duration of postoperative admission (for inclusion in online publication only).

	Hazard ratio (95% CI)	*χ* ^2^	*P*
Albumin	1.044 (1.012 to 1.076)	7.573	**0.006**
Anastomotic leak (Y)	0.127 (0.042 to 0.388)	13.150	**<0.001**
Stoma (Y)	0.609 (0.437 to 0.848)	8.613	**0.003**
Lymph node ratio	0.264 (0.102 to 0.686)	7.479	**0.006**

## What Does This Paper Add to the Literature?

We present a number of previously undescribed variables associated with delay to adjuvant chemotherapy. We show that extended postoperative stay is related to delay to commencement of adjuvant chemotherapy.
